# Routing Brain Traffic Through the Von Neumann Bottleneck: Parallel Sorting and Refactoring

**DOI:** 10.3389/fninf.2021.785068

**Published:** 2022-03-01

**Authors:** Jari Pronold, Jakob Jordan, Brian J. N. Wylie, Itaru Kitayama, Markus Diesmann, Susanne Kunkel

**Affiliations:** ^1^Institute of Neuroscience and Medicine (INM-6) and Institute for Advanced Simulation (IAS-6) and JARA-Institute Brain Structure-Function Relationships (INM-10), Jülich Research Centre, Jülich, Germany; ^2^RWTH Aachen University, Aachen, Germany; ^3^Department of Physiology, University of Bern, Bern, Switzerland; ^4^Jülich Supercomputing Centre, Jülich Research Centre, Jülich, Germany; ^5^RIKEN Center for Computational Science, Kobe, Japan; ^6^Department of Physics, Faculty 1, RWTH Aachen University, Aachen, Germany; ^7^Department of Psychiatry, Psychotherapy and Psychosomatics, Medical Faculty, RWTH Aachen University, Aachen, Germany; ^8^Faculty of Science and Technology, Norwegian University of Life Sciences, Ås, Norway

**Keywords:** spiking neural networks, large-scale simulation, distributed computing, parallel computing, sparsity, irregular access pattern, memory-access bottleneck

## Abstract

Generic simulation code for spiking neuronal networks spends the major part of the time in the phase where spikes have arrived at a compute node and need to be delivered to their target neurons. These spikes were emitted over the last interval between communication steps by source neurons distributed across many compute nodes and are inherently irregular and unsorted with respect to their targets. For finding those targets, the spikes need to be dispatched to a three-dimensional data structure with decisions on target thread and synapse type to be made on the way. With growing network size, a compute node receives spikes from an increasing number of different source neurons until in the limit each synapse on the compute node has a unique source. Here, we show analytically how this sparsity emerges over the practically relevant range of network sizes from a hundred thousand to a billion neurons. By profiling a production code we investigate opportunities for algorithmic changes to avoid indirections and branching. Every thread hosts an equal share of the neurons on a compute node. In the original algorithm, all threads search through all spikes to pick out the relevant ones. With increasing network size, the fraction of hits remains invariant but the absolute number of rejections grows. Our new alternative algorithm equally divides the spikes among the threads and immediately sorts them in parallel according to target thread and synapse type. After this, every thread completes delivery solely of the section of spikes for its own neurons. Independent of the number of threads, all spikes are looked at only two times. The new algorithm halves the number of instructions in spike delivery which leads to a reduction of simulation time of up to 40 %. Thus, spike delivery is a fully parallelizable process with a single synchronization point and thereby well suited for many-core systems. Our analysis indicates that further progress requires a reduction of the latency that the instructions experience in accessing memory. The study provides the foundation for the exploration of methods of latency hiding like software pipelining and software-induced prefetching.

## 1. Introduction

Over the last two decades, simulation algorithms for spiking neuronal networks have continuously been improved. The largest supercomputers available can be employed to simulate networks with billions of neurons at their natural density of connections. The respective codes scale well over orders of magnitude of network size and number of compute nodes (Jordan et al., 2018). Still, simulations at the brain scale are an order of magnitude slower than real time, hindering the investigation of processes such as plasticity and learning unfolding over hours and days of biological time. In addition, there is a trend of aggregating more compute power in many-core compute nodes. This further reduces the strain on inter-node communication as one limiting component but increases the urgency to better understand the fundamental operations required for routing spikes within a compute node.

The spiking activity in mammalian neuronal networks is irregular, asynchronous, sparse, and delayed. Irregular refers to the structure of the spike train of an individual neuron. The intervals between spike times are of different lengths and unordered as if drawn from a random process. Consequently, the number of spikes in a certain time interval also appears random. Asynchronous means that the spikes of any two neurons occur at different times and exhibit low correlation. The activity of neurons is sparse in time as compared to the time constants of neuronal dynamics; only few spikes are emitted in any second of biological time. Last, there is a biophysical delay in the interaction between neurons imposed by their anatomy. The delay may be a fraction of a millisecond for neurons within a distance of a few micrometers but span several milliseconds for connections between brain regions (refer to Schmidt et al., 2018a, for an example compilation of parameters).

The existence of a minimal delay in a network model together with the sparsity of spikes has suggested a three-phase cycle for an algorithm directly integrating the differential equations of the interacting model neurons (Morrison et al., 2005). First, communication between compute nodes occurs synchronously in intervals of minimal delay. This communication transmits all the spikes that have occurred on a compute node since the last communication step to the compute nodes harboring target neurons of these spikes. Second, the received spikes are delivered to their target neurons and placed in spike ring buffers representing any remaining individual delay. Finally, the dynamical state of each neuron is propagated for the time span of the minimal delay while the ring buffer is rotating accordingly. Once all neurons are updated, the next communication is due and the cycle begins anew.

Progress in each update phase is shaped by the spiking interaction between neurons and independent of the level of detail of the individual model neurons constituting the network. The choice of the neuron model, however, influences the distribution of computational load across the phases of the simulation. Some studies require neuron models with thousands of electrical compartments (Markram et al., 2015), and efficient simulation codes are available for this purpose (Carnevale and Hines, 2006; Akar et al., 2019; Kumbhar et al., 2019). Here, we focus on simulation code for networks of model neurons described by a handful of differential equations as widely used in the computational neuroscience community. These investigations range from studies with several thousands of neurons on the fundamental interplay between excitation and inhibition (Brunel, 2000) to models attempting to capture the natural density of wiring (Potjans and Diesmann, 2014; Billeh et al., 2020) and the interaction between multiple cortical areas (Joglekar et al., 2018; Schmidt et al., 2018b). Previous measurements on a production code (Jordan et al., 2018) already show that for networks of such simple model neurons the dominating bottleneck for further speed-up is neither the communication between computes nodes nor the update of the dynamical state of the neurons, but the spike-delivery phase. The empirical finding is elegantly confirmed by an analytical performance model encompassing different types of network and neuron models (Cremonesi and Schürmann, 2020; Cremonesi et al., 2020). These authors further identify the latency of memory access as the ultimate constraint of the spike-delivery phase.

Profiling tools like Intel VTune provide measures on where an application spends its time and how processor and memory are used. Two basic measures are the total number of instructions carried out and the number of clock ticks the processor required per instruction (CPI). The former characterizes the amount of computations that need to be done to arrive at the solution. The latter describes how difficult it is on average to carry out an individual instruction due to the complexity of the operation and the waiting for accessing the corresponding part of memory. The product of the two measures is the total number of clockticks and should correlate to the wall clock time required to complete the simulation phase under investigation. Methods of software pipelining and software-induced prefetching attempt to improve the CPI by better vectorization of the code or by indicating to the processor which memory block will soon be required. These optimizations may lead to an increase in the actual number of instructions but as long as the product with the reduced CPI decreases, performance is improving. Nevertheless, a low CPI does not mean that the code is close to optimal performance. If the code is overly complicated, for example by recalculating known results or missing out on regularities in the data it may underutilize data that has been retrieved from memory rendering advanced methods of optimization fruitless. Therefore, in the present study, as a first step, we do not consider pipelining and prefetching but exclusively assess the number of instructions required by the algorithm. It turns out that a better organized algorithm indeed avoids unnecessary tests and indirections. This decrease in the number of instructions also decreases CPI as a side effect until with increasing sparseness of the network CPI climbs up again. The control flow in the code becomes more predictable for the processor until the fragmentation of memory limits the success. The results of our study give us some confidence that further work can now directly address improving the CPI.

In Section 2, we expose spike delivery as the present bottleneck for the simulation of mammalian spiking neuronal networks, characterize analytically the transition to sparsity with growing network size, and present the original algorithm as well as state-of-the-art performance data. Next, we introduce the software environment of our study and the neuronal network model used to obtain quantitative data (Section 3). On the basis of these preparatory sections, Section 4 presents a new algorithm streamlining the routing of spikes to their targets. Subsequently, Section 5 evaluates the success of the redesign and identifies the origin of the improvement by profiling. Finally, Section 6 embeds the findings into the ongoing efforts to develop generic technology for the simulation of spiking neuronal networks.

The conceptual and algorithmic work described here is a module in our long-term collaborative project to provide the technology for neural systems simulations (Gewaltig and Diesmann, 2007). Preliminary results have been presented in abstract form (Kunkel, 2019).

## 2. Spike Delivery as Memory-Access Bottleneck

The temporally sparse event-based communication between neurons presents a challenging memory-access bottleneck in simulations of spiking neuronal networks for modern architectures optimized for dense data. In the neuronal simulator NEST (Section 3.1), which we use as reference implementation in this study, delivery of spikes to their synaptic and neuronal targets involves frequent access to essentially random memory locations, rendering automatic prediction difficult and leading to long data-access times due to ineffective use of caches. The following subsection provides an analysis of the sparsity of the network representation for increasing numbers of Message Passing Interface (MPI) processes and threads. Based on this, there follows a description of the connection data structures and spike-delivery algorithm in the original implementation. The final subsection provides example benchmarking data for this state-of-the-art simulation code.

### 2.1. Sparsity of Network Representation

We consider a network of *N* neurons distributed in a round-robin fashion across *M* MPI processes and *T* threads per process. Each neuron receives *K* incoming synapses, which are represented on the same thread as their target neuron. In a weak scaling scenario, the computational load per process is kept constant. This implies that the number of thread-local synapses


(1) $$\begin{array}{r} S=NK/\left(MT\right) \end{array}$$

does not change. The total network size, in contrast, increases with *MT*. In the limit of large network sizes, each synapse on a given thread originates from a different source neuron. This scenario was already considered (Kunkel et al., 2014, section 2.4) at the time to analyze the increase in memory overhead observed with increasing sparsity. For completeness, we briefly restate this result in the parameters used in the present work.

The probability that a synapse has a particular neuron *j* as source neuron is 1/*N* and, conversely, the probability that the synapse has a different source neuron is 1−1/*N*. The probability that none of the *S* thread-local synapses has neuron *j* as a source is $p_{\emptyset }={\left(1-1/N\right)}^{S}$. Conversely, *p* = 1−*p*_∅_ denotes the probability that *j* is the source to at least one of the thread-local synapses. Therefore, the expected number of unique source neurons of the thread-local synapses are given by *N*_u_ = *pN* expanding to


(2) $$\begin{array}{r} N_{\text{u}}=\left(1-{\left[{\left(1-\frac{1}{N}\right)}^{N}\right]}^{\frac{K}{MT}}\right)N \end{array}$$

which is Equation (6) of Kunkel et al. (2014). In weak scaling, *MT* grows proportionally to *N* such that


$$N_{\text{u}}=\left(1-{\left[{\left(1-\frac{1}{N}\right)}^{N}\right]}^{\frac{S}{N}}\right)N$$

where they further identified the term [·] in the limit of large *N* as the definition of the exponential function with argument −1 and therefore


$${\tilde{N}}_{\text{u}}=\left(1-\exp\left(-\frac{S}{N}\right)\right)N.$$

They confirm that the limit of *N*_u_ is indeed *S* and that a fraction ζ of *S* is reached at a network size of


(3) $$\begin{array}{r} N_{\zeta }=\frac{S}{2\left(1-\zeta \right)}. \end{array}$$

**Figure 1:**
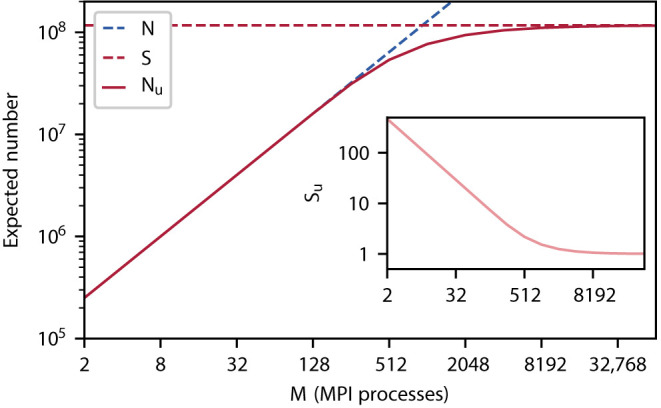
Expected number of unique source neurons *N*_u_ (pink curve) of all thread-local synapses as a function of the number of the MPI processes *M* assuming *T* = 12 threads and 125, 000 neurons per MPI process in a weak-scaling scenario; the total number of neurons *N* (dashed blue curve) and number of thread-local synapses *S* (dashed pink horizontal line for *K* = 11, 250 synapses per neuron). Inset: Expected number of thread-local synapses per unique source neuron *S*_u_ = *S*/*N*_u_ (light pink curve). All graphs in double logarithmic representation.

Figure 1 illustrates the point where in weak scaling the total network size *N* equals the number of thread local synapses *S*. Here, the number of unique source neurons *N*_u_ of the thread-local synapses bends. According to the definition (1) of *S*, here a particular target neuron chooses its *K* incoming synapses from the same total number of threads *MT* = *K* and already half ($\zeta =\frac{1}{2}$) of the source neurons of the thread-local synapses are unique. The number of thread-local synapses per unique source neuron *S*_u_ indicates the sparsity of the network representation on a compute node (inset of Figure 1). The measure converges to one exhibiting a bend at the same characteristic point as *N*_u_.

### 2.2. Memory Layout of Synapse and Neuron Representations

**Figure 2:**
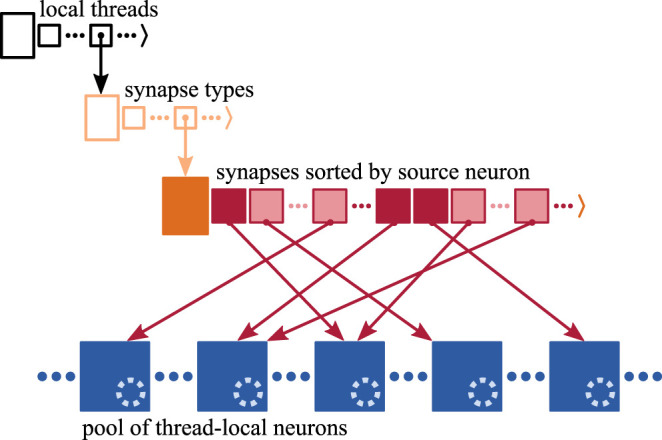
Memory layout of synapse and neuron representations on each MPI process. Each process stores the local synapses (pink filled squares) in a three-dimensional resizable array sorted by hosting thread and synapse type. At the innermost level, synapses are arranged in source-specific target segments (dark pink: first synapse; light pink: subsequent targets); only one innermost array is shown for simplicity. Target neurons (blue filled squares) are stored in neuron-type and thread-specific memory pools; only one pool is shown for simplicity. Each neuron maintains a spike ring buffer (dotted light blue circles). Synapses have access to their target neurons through target identifiers (dark pink arrows).

A three-dimensional resizable array stores the process-local synapses sorted by hosting thread and synapse type (Figure 2), where synapses are small in size, each typically taking up few tens of Bytes. Each synapse has access to its target neuron, which is hosted by the same MPI process and thread (Morrison et al., 2005). The target identifier provides access either through a pointer to the target neuron consuming 8 B or an index of 2 B that is used to retrieve the corresponding pointer. Here, we use the latter implementation of the target identifier reducing per-synapse memory usage at the cost of an additional indirection (refer to Section 3.3.2 in Kunkel et al., 2014).

In the innermost arrays of the data structure, synapses are sorted by source neuron, which is an optimization for small to medium scale systems (see Section 3.3 in Jordan et al., 2018) exploiting that each neuron typically connects to many target neurons (out-degree). Thereby, synapses are arranged in target segments, each consisting of at least one target synapse potentially followed by subsequent targets (Section 2.1). In a weak-scaling experiment, the increasing sparsity of the network in the small to medium scale regime (Section 2.1) influences the composition of the innermost array. As synapses are to an increasing degree distributed across MPI processes and threads, the expected number of source-specific target segments increases while the average segment size decreases (cf. *N*_u_ and *S*_u_ in Figure 1, respectively). Note that the degree of distribution also depends on the number of synapse types, which is however not considered in this study.

A model neuron easily takes up more than a Kilobyte of memory. Multi-chunk memory pools enable contiguous storage of neurons of the same type hosted by the same thread, where due to the many-to-one relation between target synapses and neurons, the order of memory locations of target neurons is independent of the order of synapses in the target segments.

Synaptic transmission of spikes entails delays, which influence the time when spikes take effect on the dynamics of the target neurons. As typically synapses from many different source neurons converge on the same target neuron (in-degree), it is more efficient to jointly account for their delays in the neuronal target. Therefore, each neuron maintains a spike ring buffer serving as temporary storage and scheduler for the incoming spikes (Morrison et al., 2005).

### 2.3. Original Spike-Delivery Algorithm

Every time all local neurons have been updated and all recent spikes have been communicated across MPI processes, the spike data needs to be delivered from the process-local MPI receive buffers to the process-local synaptic and neuronal targets. Each spike entry is destined for an entire target segment of synapses (Section 2.2), which is an optimization for the small to medium scale regime introduced in Jordan et al. (2018). Therefore, each entry conveys the location of the target segment within the three-dimensional data structure storing the process-local synapses (Figure 2), i.e., identifiers for the hosting thread and the type of the first synapse of the target segment, as well as the synapse's index within the innermost resizable array.

In the original algorithm, each thread reads through all spike entries in the MPI receive buffer but it only proceeds with the delivery of a spike if it actually hosts the spike's targets - all spike entries indicating other hosting threads are skipped. Each thread delivers the relevant spikes to every synapse of the corresponding target segments one by one. On receiving a spike, a synapse transfers synaptic delay and weight to the corresponding target neuron, where the stored target identifier provides access to the neuron. The transmitted synaptic properties, delay and weight, define the time and amplitude of the spike's impact on the neuron, respectively, allowing the neuron to add the weight of the incoming spike to the correct position in the neuronal spike ring buffer.

In a weak-scaling experiment, the increasing sparsity of the network in the small to medium scale regime (Section 2.1) influences algorithmic progression and memory-access patterns. Access to target neurons and their spike ring buffers is always irregular regardless of the degree of distribution of the network across MPI processes, but memory access to synapses become progressively irregular. The number of spike entries communicated via MPI increases to cater to the growing number of target segments (Section 2.2). In consequence, each thread needs to process even more spike entries, delivering relevant spikes to even more but shorter target segments, where successively visited target segments are typically in nonadjacent memory locations. In the sparse limit where each target segment consists of a single synapse, spike delivery to both neuronal and synaptic targets requires accessing essentially random locations in memory. As many synapses of different source neurons converge on the same target neuron, it is impossible to arrange target neurons in memory such that their order corresponds to the order in synaptic target segments. The pseudocode in 2.3.1 summarizes this original spike-delivery algorithm.

#### 2.3.1. Pseudocode


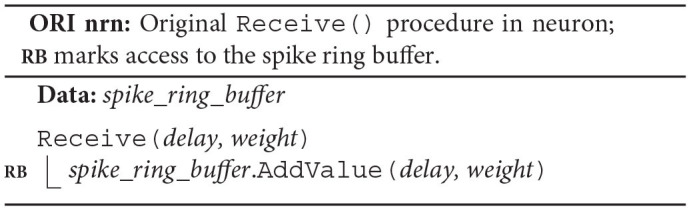



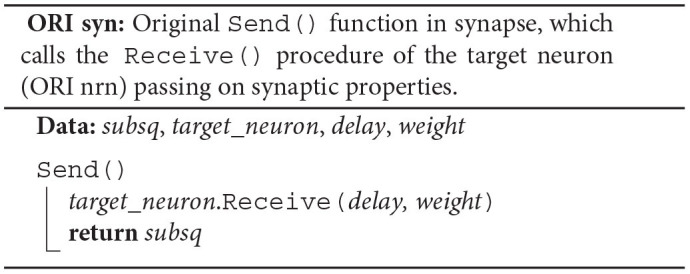



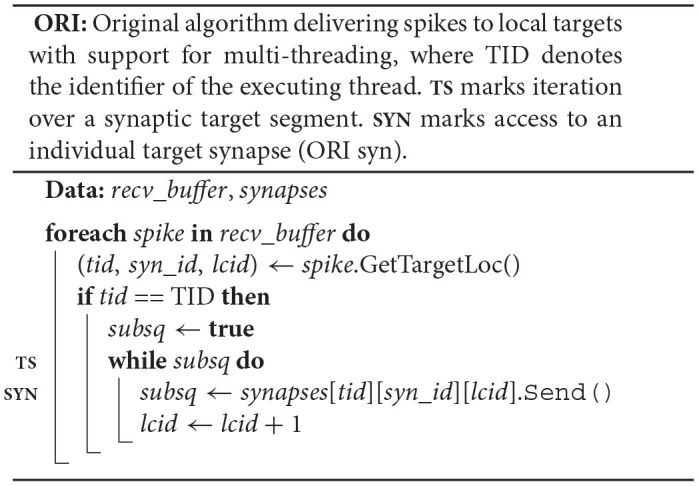


The original algorithm delivers spikes to the neuronal spike ring buffers through the target synapses. Each neuron owns a *spike*_*ring*_*buffer*, where the neuron member function Receive() triggers the spike delivery by calling the spike ring buffer member function AddValue(), which then adds the weight of the spike to the correct position in the buffer (ORI nrn; **RB**). To this end, both Receive() and AddValue() require the synaptic properties *delay* and *weight*.

Each synapse stores properties such as *delay* and *weight*, an identifier enabling access to the target neuron (*target*_*neuron*), and an indicator (*subsq*) of whether the target segment continues or not (ORI syn). The synapse member function Send() calls the member function Receive() of the target neuron passing on the synaptic properties and returns the indicator *subsq*.

The original spike-delivery algorithm has access to the MPI spike-receive buffer (*recv*_*buffer*) containing all spike entries that need to be delivered and to a three-dimensional resizable array of process-local *synapses* ordered by hosting thread and synapse type (ORI; see Figure 2). For each spike entry, the 3D location of the first target synapse is extracted and assigned to the variables *tid*, *syn*_*id*, and *lcid*, which indicate hosting thread, synapse type, and location in the innermost *synapses* array, respectively. If the executing thread (TID) is the hosting thread of the target synapse, then the variable *lcid* is used in the enclosed while loop to iterate over the spike's entire synaptic target segment within the innermost array *synapses*[*tid*][*syn*_*id*] (**TS**). To deliver a spike to the target synapse at position *lcid*, the synapse member function Send() is called on *synapses*[*tid*][*syn*_*id*][*lcid*] returning the indicator *subsq* (**SYN**).

### 2.4. Simulation Time

The work of Jordan et al. (2018) shows that spike delivery is the dominating phase of simulation time from networks with a few hundred thousand neurons to the regime of billions of neurons. In the latter, the number of neurons in the network exceeds the number of synapses represented on an individual compute node; each synapse on a given compute node has a unique source neuron (Section 2.1). Therefore, a neuron finds either a single target neuron on a compute node or none at all. Assuming a random distribution of neurons across MPI processes, the network is fully distributed in terms of its connectivity. From this point on, the computational costs of spike delivery on a compute node do not change with growing network size in a weak scaling scenario; each synapse receives spikes with a certain frequency and all spikes come from different sources. What is still increasing are the costs of communication between the compute nodes. Nevertheless, for smaller networks below the limit of sparsity, Jordan et al. (2018) provide optimizations (their section 3.3) exploiting the fact that a spike finds multiple targets on a compute node. This reduces both communication time and spike-delivery time, but the effect vanishes in the limit (as shown in Figure 7C in Jordan et al., 2018; 5g-sort) where the code continues to scale well with the maximal but invariant costs of spike delivery.

The network model of Jordan et al. (2018) exhibits spike-timing dependent plasticity in its synapses between excitatory neurons. The spike-delivery phase calculates the plastic changes at synapses because synaptic weights only need to be known when a presynaptic spike is delivered to its target (Morrison et al., 2007a). Depending on the specific plasticity rule, these computations may constitute a considerable fraction of the total spike delivery time. Therefore, from the data of Jordan et al. (2018), we cannot learn which part of the spike-delivery time is due to the calculation of synaptic plasticity and which part is due to the actual routing of spikes to their targets. In order to disentangle these contributions, the present study uses the same network model but considers all synapses as static (Section 3.2).

**Figure 3:**
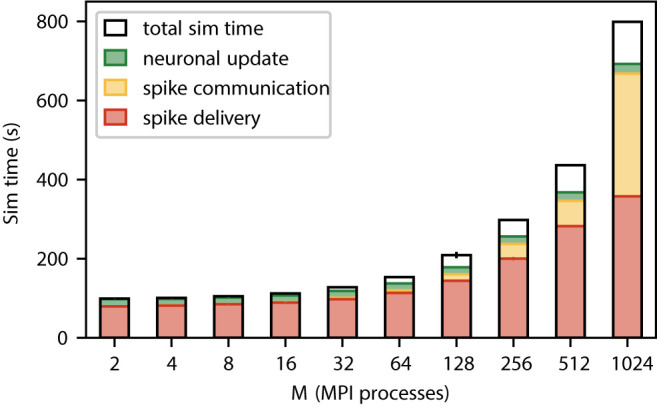
Contributions to the simulation time (sim time) for spiking neural network simulations with NEST (Section 3.1) where the number of MPI processes increases proportionally with the total number of neurons. Weak-scaling experiment running 2 MPI processes per compute node and 12 threads per MPI process, with a workload of 125, 000 neurons per MPI process (network model see Section 3.2). The network dynamics is simulated for 1 s of biological time; spikes are communicated across MPI processes every 1.5 ms. Time is spent on spike delivery (red bars), communication of spike data (yellow bars), neuronal update (green bars), and total sim time (black outline). Error bars (for most numbers of MPI processes hardly visible) indicate the SD over three repetitions. Timings obtained via manual instrumentation of the respective parts of the source code, measured on JURECA CM (Section 3.3).

Figure 3 shows a weak scaling of our static neuronal network model across the critical region where sparsity has not yet reached the limit. This confirms that even in the absence of synaptic plasticity spike delivery is the dominant contribution to simulation time independent of the number of MPI processes. The network on a single MPI process roughly corresponds to the smallest cortical network in which the natural number of synapses per neuron and the local connection probability of 0.1 can simultaneously be realized (Potjans and Diesmann, 2014). While our weak scaling conserves the former quantity, the latter drops. In the regime from 2 to 512 MPI processes, the absolute time required for spike delivery almost quadruples (factor of 3.9). Beyond this regime, the relative contribution of spike delivery to simulation time drops below 50% because the time required for communication is increasing. The absolute time for neuronal update remains unchanged throughout as the number of neurons per MPI process is fixed.

## 3. Benchmarking Framework

### 3.1. Simulation Engine

Over the past two decades, simulation tools in computational neuroscience have increasingly embraced a conceptual separation of generic simulation engines and specific models of neuronal networks (Einevoll et al., 2019). Many different models can thus be simulated with the same simulation engine. This enables the community to separate the life cycle of a simulation engine from those of specific individual models and to maintain and further develop simulation engines as an infrastructure. Furthermore, this separation is useful for the cross-validation of different simulation engines.

One such engine is the open-source community code NEST^1^ (Gewaltig and Diesmann, 2007). The quantitative analysis of the state-of-the-art in the present study is based on this code and alternative concepts are evaluated in its software framework. This ensures that ideas are immediately exposed to the complications and legacy of real-world code. NEST uses a hybrid between an event-driven and a time-driven simulation scheme to exploit that individual synaptic events are rare whereas the total number of spikes arriving at a neuron is large (Morrison et al., 2005). Neurons are typically updated every 0.1 ms and spike times are constrained to this time grid. For high-precision simulations, spikes can also be processed in continuous time (Morrison et al., 2007b; Hanuschkin et al., 2010). In contrast, synapses are only updated when a spike is arriving from the corresponding presynaptic neuron. The existence of a biophysical delay in the spiking interaction between neurons enables a global exchange of spike data between compute nodes in intervals of minimal delay. The data structures and algorithms for solving the equations of neuronal networks of natural size (Morrison and Diesmann, 2008; Helias et al., 2012; Kunkel et al., 2012, 2014; Jordan et al., 2018) as well as technology for network creation (Ippen et al., 2017) and the language interface (Eppler et al., 2009; Zaytsev and Morrison, 2014; Plotnikov et al., 2016) are documented and discussed in the literature in detail. For the purpose of the present study, it suffices to characterize the main loop of state propagation (Section 1) and concentrate on the details of spike delivery (Section 2).

Besides spikes, NEST supports gap junctions (Hahne et al., 2015; Jordan et al., 2020) as a further biophysical mechanism of neuronal interaction. To allow modeling of mechanisms affecting network structure on longer time scales, NEST implements models of neuromodulated synaptic plasticity (Potjans et al., 2010), voltage-dependent plasticity (Stapmanns et al., 2021), and structural plasticity (Diaz-Pier et al., 2016). For the representation of more abstract network models, NEST, in addition, supports binary neuron models (Grytskyy et al., 2013) and continuous neuronal coupling (Hahne et al., 2017) for rate-based and population models.

The present work is based on commit 059fe89 of the NEST 2.18 release.

### 3.2. Network Model

As earlier studies on neuronal network simulation technology (latest Jordan et al., 2018), we use a generic model of mammalian neuronal networks (Brunel, 2000) for measuring and comparing proposed algorithmic modifications. The model description and parameters are given in parameter Tables 1–3 of Jordan et al. (2018), and Section 2.4 gives an overview of performance for state-of-the-art code. A figure illustrating the structure of the model is part of the NEST user-level documentation^2^. The sole difference of the investigated model with respect to previous studies is the restriction to static synapses for excitatory-excitatory connections. These synapses have a fixed weight whereas in former studies they exhibited spike-timing dependent plasticity (Morrison et al., 2007a).

The network is split into two populations: excitatory (80%) and inhibitory neurons (20%). These are modeled by single-compartment leaky-integrate-and-fire dynamics with alpha-shaped postsynaptic currents. Parameters are homogeneous across all neurons. Each neuron receives a fixed number of excitatory and inhibitory connections with presynaptic partners randomly drawn from the respective population. Thus, every neuron has 11, 250 incoming and, on average, 11, 250 outgoing synapses, independent of the network size. Inhibitory synapses are stronger than excitatory synapses to ensure the stability of the dynamical state of the network. The simulation of 10 ms of biological time, called the init phase, is followed by the further simulation of 1 s of biological time. The former initiates the creation and initialization of data structures that are unchanged in the simulation of subsequent time stretches. The measured wall-clock time of the latter, called the simulation phase, is referred to as “sim time.” The mean firing rate across all network sizes considered in this study is 7.56 Hz with a SD of 0.1 Hz.

### 3.3. Systems

The JURECA Cluster Module (JURECA CM) and the K computer are already specified in Jordan et al. (2018), their characteristics are repeated here in the same words for completeness except the renaming of JURECA to JURECA CM after the addition of a booster module not used here. JURECA CM (Krause and Thörnig, 2018) consists of 1,872 compute nodes, each housing two Intel Xeon E5-2680 v3 Haswell CPUs with 12 cores each at 2.5 GHz for a total of 1.8 PFLOPS. Most of the compute nodes have 128 GB of memory available. In addition, 75 compute nodes are equipped with two NVIDIA K80 GPUs, which, however, are not used in this study. The nodes are connected via Mellanox EDR Infiniband.

Dynamical Exascale Entry Platform-Extreme Scale Technologies (DEEP-EST)^3^ is an EU project exploring the usage of modular supercomputing architectures. Among other components, it contains a cluster module (DEEP-EST CM) tuned for applications requiring high single-thread performance and a modest amount of memory. The module consists of one rack containing 50 nodes, each node hosting two Intel Xeon Gold 6146 Skylake CPUs with 12 cores each. The CPUs run at 3.2 GHz and have 192 GB RAM. In total, the system has 45 TFLOPS and aggregates 45 TB of main memory. The system uses Mellanox InfiniBand EDR (100 GBps) with fat tree topology for communication.

Both on JURECA CM and DEEP-EST CM, we compile the application with OpenMP enabled using GCC and link against ParaStationMPI for MPI support. In our benchmarks, to match the node architecture, we launch 2 MPI processes each with 12 threads on every node and bind the MPI processes to sockets using --cpu_bind=sockets to ensure that the threads of each process remain on the same socket.

The K computer (Miyazaki et al., 2012) features 82, 944 compute nodes, each equipped with an 8-core Fujitsu SPARC64 VIIIfx processor operating at 2 GHz, with 16 GB RAM per node, leading to a peak performance of about 11.3 PFLOPS and a total of 1,377 TB of main memory. The compute nodes are interconnected via the “Tofu” (“torus connected full connection”) network with 5 GBps per link. The K computer supports hybrid parallelism with OpenMP (v3.0) at the single node level and MPI (v2.1) for inter-node communication. Applications are compiled with the Fujitsu C/C++ Compiler and linked with Fujitsu MPI. Each node runs a single MPI process with 8 threads.

### 3.4. Software for Profiling and Workflow Management

Optimizing software requires the developer to identify critical sections of the code and to guarantee identical initial conditions for each benchmark. This is all the more true in the field of simulation technology for spiking neuronal networks. Despite the advances (Schenck et al., 2014; Cremonesi, 2019; Cremonesi and Schürmann, 2020; Cremonesi et al., 2020) in the categorization of neuronal network applications and the identification of bottlenecks, performance models are not yet sufficiently quantitative and fundamental algorithms and data structures are evolving. Therefore, the field still relies on exploration and quantitative experiments. The present work employs the profiling tool VTune to guide the development as well as the benchmarking environment JUBE for workflow management. In addition, the NEST code contains manual instrumentation to gather the cumulative times spent in the update, communicate, and deliver phases and to determine the total simulation time.

#### 3.4.1. VTune

VTune Profiler^4^ is a proprietary performance analysis tool developed by the company Intel providing both a graphical user interface and a command-line interface. It collects performance statistics across threads and MPI processes while the application is running. VTune supports different analysis types instructing the profiling program executing the application to focus on specific characteristics. From the rich set of statistical measures, we select only three basic quantities: Instructions Retired, Clockticks, and Clockticks per Instructions Retired (CPI). The Instructions Retired show the total number of completed instructions, while the CPI is the ratio of unhalted processor cycles (clockticks) relative to the number of instructions retired indicating the impact of latency on the application's execution.

#### 3.4.2. JUBE

Documenting and reproducing benchmarking data requires the specification of metadata on the computer systems addressed and metadata on the configurations for compiling the application, for running the simulations, and for evaluating the results. The Jülich Benchmarking Environment (JUBE) ^5^(Lührs et al., 2016) is a software suite developed by the Jülich Supercomputing Centre. We employ JUBE to represent all metadata of a particular benchmark by a single script.

## 4. Redesign of Spike-Delivery Algorithm

The algorithmic redesign concentrates on the initial part of spike delivery and access to the spike ring buffers. The initial part of the original algorithm (Section 2.3) does not fully parallelize the sorting of spike events according to the target thread (Section 4.1). Furthermore, access to the spike ring buffers is hidden from the algorithm as the buffer is considered an implementation detail of the object representing a neuron (Section 4.2). Acronyms given in the titles of the subsections label the specific modifications for brevity and serve as references in pseudocode and figures.

### 4.1. Streamlined Processing of Spike Entries (SRR)

In the original spike-delivery algorithm (Section 2.3), each thread needs to read all spike entries in the MPI receive buffer, even those not relevant for its thread local targets, causing an overhead per spike entry, and hence per process-local synaptic target segment. Moreover, for each relevant spike entry, the thread hosting the targets needs to identify the correct innermost array in the three-dimensional resizable array storing the process-local synapses (Figure 2) based on the synapse-type information provided by the spike entry. This entails additional per target-segment overhead.

To address these issues, we adapt the original spike-delivery algorithm such that instead of directly dispatching the data from the receive buffer to the thread-local targets, we introduce a two-step process: First, the threads sort the spike entries by hosting thread and synapse type in parallel, and only then the threads dispatch the spikes, now exclusively reading relevant spike entries. To this end, we introduce a new data structure of nested resizable arrays, called spike-receive register (SRR), where each thread is assigned its own domain for writing. After each spike communication, a multi-threaded transfer of all spike entries from the MPI receive buffer to the spike-receive register takes place: each thread reads a different section of the entire receive buffer and transfers the entries to their SRR domains. The domains are in turn organized into separate resizable arrays, one per hosting thread. Nested resizable arrays enable the further sorting by synapse type. In this way, each element of the MPI receive buffer is read only once and spike entries are immediately sorted. This allows for a subsequent multi-threaded delivery of spikes from the spike-receive register to the corresponding target synapses and neurons such that all spike entries are exclusively read by their hosting thread. At this point, all a hosting thread has to do is to sequentially work through every resizable array exclusively prepared for it in the sorting phase. The additional sorting by synapse type allows the hosting thread to deliver all spikes targeting synapses of the same type in one pass.

### 4.2. Exposure of Code Dependencies (P2RB)

In the original spike-delivery algorithm (Section 2.3), the target synapse triggers the delivery of a spike to its target neuron, which then adds the spike to its spike ring buffer. For the entire spike-delivery process, this results in alternating access to target synapses and target neurons, or more precisely, the target neurons' spike ring buffers. As synapses store the target identifiers and other relevant information, access to a target synapse is a precondition for access to its target neuron.

In order to expose this code dependency, we separate the two delivery steps: spike delivery to target synapse and corresponding target neuron are now triggered sequentially at the same call-stack level. Moreover, instead of storing a target identifier, each synapse now stores a pointer to the target neuron's spike ring buffer allowing for direct access when delivering a spike. Therefore, the quantitative analysis (Section 5.1) refers to this set of optimizations as P2RB as an acronym for “pointer to ring buffer”.

### 4.3. Pseudocode


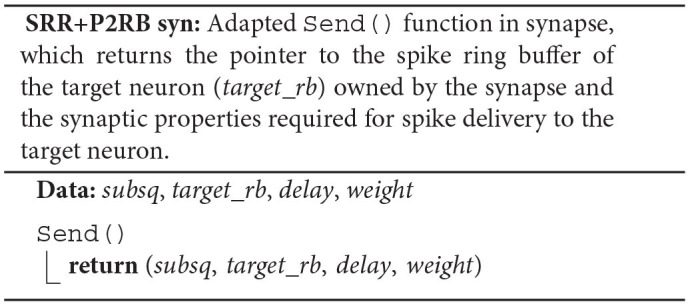


The pseudocode SRR+P2RB illustrates the changes to the original spike-delivery algorithm (ORI) resulting from the two new algorithms SRR (Section 4.1) and P2RB (Section 4.2).

Instead of a target-neuron identifier, each synapse now owns a pointer (*target*_*rb*) to the neuronal spike ring buffer. The synapse member function Send() returns the pointer and the synaptic properties *delay* and *weight* in addition to the indicator *subsq* (SRR+P2RB syn). This allows the algorithm to directly call AddValue() on the spike ring buffer (SRR+P2RB; **RB**) after the call to the synapse member function Send() (**SYN**). The Receive() member function of the target neuron (ORI nrn) is no longer required. Additionally, the algorithm now makes use of a spike receive register (*spike*_*reg*) for a preceding thread-parallel sorting of the spike entries from the MPI receive buffer by hosting thread (*tid*) and synapse type (*syn*_*id*), where each thread writes to its private region of the register (*spike*_*reg*[TID]). Spikes are then delivered from the spike receive register instead of the MPI receive buffer, where each thread processes only those regions of the register that contain spike entries for thread-local targets (*spike*_*reg*[*tid*][TID] for all possible *tid*).


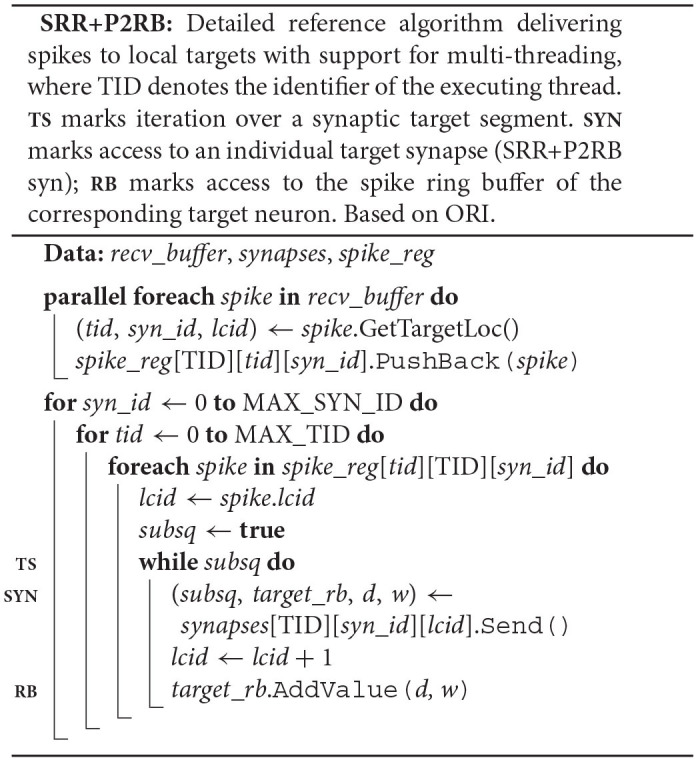


## 5. Results

The new data structures and algorithms of Section 4 can be combined because they modify different parts of the code. As the efficiency of the optimizations may depend on the hardware architecture, we assess their performance on three computer systems (Section 5.1). Subsequently, we investigate in Section 5.2 the origin of the performance gain by evaluating the change in the total number of instructions required and the average number of clockticks consumed per instruction.

### 5.1. Effect of Redesign on Simulation Time

**Figure 4:**
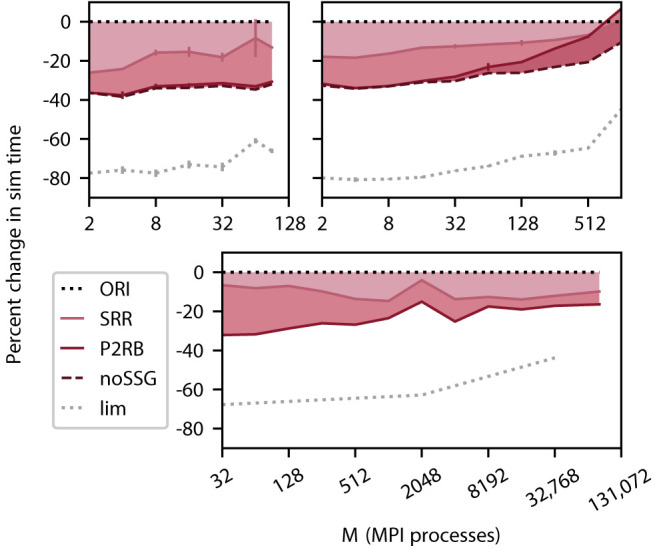
Cumulative relative change in simulation time after a redesign of spike delivery as a function of the number of MPI processes *M*. Top left panel DEEP-EST CM and top right panel JURECA CM: linear-log representation for a number of MPI processes *M*∈{2; 4; 8; 16; 32; 64; 90} and *M*∈{2; 4; 8; 16; 32; 64; 128; 256; 512; 1024}, respectively. Weak scaling of benchmark network model with the same configuration as in Figure 3; error bars show SD based on 3 repetitions. Bottom panel K computer: number of MPI processes *M*∈{32; 64; 128; 256; 512; 1024; 2048; 4096;  8192; 16, 384; 32, 768; 82, 944}, gray dotted curve: *M*∈{32; 2048;  32, 768}. Weak scaling with different configurations (1 MPI process per compute node; 8 threads per MPI process; 18, 000 neurons per MPI process). The black dotted line at zero indicates the performance of the original code (ORI, Section 2.3). The light carmine red curve indicates a change in sim time (shading fills area to reference) due to sorting of spike entries prior to delivery (SRR, Section 4.1). The dark carmine red curve indicates an additional change in sim time due to providing synapses with direct pointers to neuronal spike ring buffers (P2RB, Section 4.2). The dashed brown curve shows an additional change in sim time after removal of an unrequired generic function call (noSSG). Gray dotted curve indicates a hypothetical limit to the decrease in sim time assuming spike delivery takes no time.

We select three computer systems for their differences in architecture and size (Section 3.3) to measure simulation times for a weak scaling of the benchmark network model (Section 3.2). The number of neurons per MPI process is significantly larger on the DEEP-EST CM and the JURECA CM (125, 000) than on the K computer (18, 000) making use of the respectively available amount of memory per process. On all three systems, we observe a relative reduction in simulation time by more than 30% (Figure 4) for the combined optimizations compared to the original code (ORI, Section 2.3). This includes the removal of a call to a function named set_sender_gid() from the generic spike delivery code (noSSG). This function attaches identifying information about the source of the corresponding spike which is only required by specific non-neuronal targets such as recorders. However, it causes per-target-segment overhead in all simulations. The functionality can hence be moved to a more specialized part of the code, e.g., the recorder model, and thereby regained if required. The DEEP-EST CM hardly benefits from the removal of the call but the batchwise processing of target segments has an increasing gain reaching 20% at 90 MPI processes. On JURECA CM, the function call does limit the performance and its removal alone improves the performance by 20% for large numbers of MPI processes. Across systems and a number of MPI processes, the combined optimizations lead to a sustained reduction in simulation time.

The new data structures and algorithms address the spike-delivery phase only, but an optimization can only reduce simulation time to the extent the component of the code to be optimized contributes to the total time consumed as indicated by the limiting curve in Figure 4. In the neuronal network simulations considered here, the delivery of spikes from MPI buffers to their targets consumes the major part of simulation time. Initially, spike delivery takes up 80% of the simulation time for the DEEP-EST CM and the JURECA CM and 70% for the K computer, but on all three systems, the relative contribution decreases with an increasing number of MPI processes. Under weak scaling into regimes beyond 1, 024 MPI processes, the absolute time required for spike delivery also initially grows but converges as the expected number of thread-local targets per spike converges to one (cf. Jordan et al., 2018). Although spike-delivery time increases throughout the entire range of MPI processes on DEEP-EST CM and JURECA CM, the relative contribution to simulation time declines because the time required by communication between MPI processes increase more rapidly (for JURECA CM data cf. Figure 3).

P2RB increases the size of synapse objects by introducing an 8 B pointer to the neuronal spike ring buffer replacing the 2 B local neuron index of the original algorithm (Section 2.3). We hypothesize that this increase underlies the declining success and ultimately disadvantageous effect observed on JURECA CM. Control simulations using the original code but with an artificially increased object size confirm this hypothesis (data not shown).

### 5.2. Origin of Improvement

The new data structures and algorithms realize a more fine-grained parallelization and avoid indirections in memory accesses (Section 4). These changes significantly speed up the application (Figure 4) across architectures and network sizes. In order to understand the origin of this improvement, we employ the profiling tool VTune (Section 3.4.1) which gives us access to the CPU's microarchitectural behavior. In the analysis, we concentrate on the total number of instructions executed and the clockticks per instruction retired (CPI).

The total number of instructions decreases by close to 50% on all scales. Nevertheless, the contribution of noSSG to the reduction in the number of instructions becomes larger as this algorithm removes code which is called for every target segment. The number of target segments, however, increases with the number of MPI processes until a limit is asymptotically approached (Figure 1).

**Figure 5:**
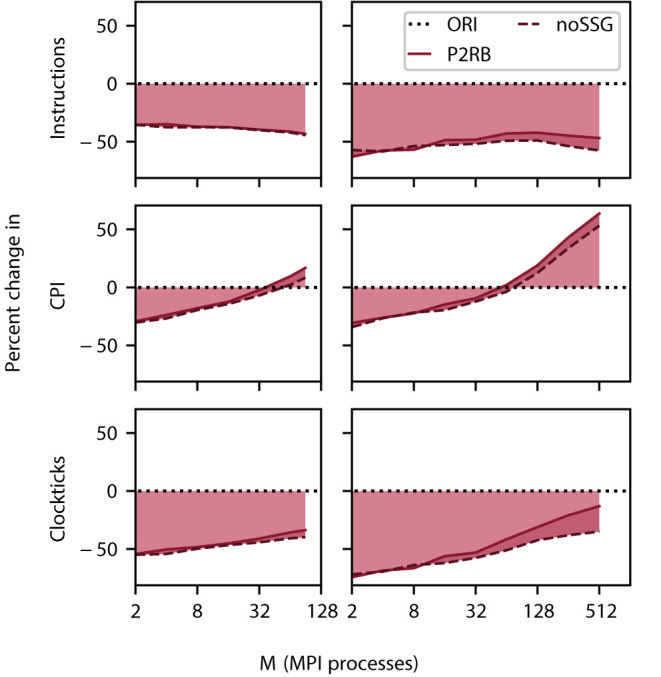
Relative change in instructions retired (top row), clockticks per instruction retired (CPI, middle), and clockticks (bottom) during spike delivery for P2RB (including SRR as in Figure 4), and noSSG as a function of the number of MPI processes. Raw data for all three quantities was obtained by VTune (Section 3.4.1). Left column DEEP-EST CM and right column JURECA CM: linear-log representation for number of MPI processes *M*∈{2; 4; 8; 16; 32; 64; 90} and *M*∈{2; 4; 8; 16; 32; 64;  128; 256; 512}, respectively. Black dotted line at zero percent (ORI, Section 2.3) indicates the performance of the original code. Weak scaling of benchmark network model as in Figure 4.

For small problem sizes, the CPI decreases when compared against the baseline (SRR+P2RB), but at around 32 MPI processes, the instructions start to consume more clockticks than in the original algorithm (Figure 5). This behavior is apparent on DEEP-EST CM as well as JURECA CM where the additional noSSG optimizations improve performance slightly. We interpret this observation as follows. Initially the more orderly organization of memory enables a shorter latency in memory access. At larger network sizes, CPI is dominated by memory access to the fragmented target segments and this dominance is more pronounced as the new code spends fewer instructions on reading the receive buffer.

Taken individually, the two metrics alone are not sufficient for explaining the decrease in simulation time (Figure 4). The product of the number of instructions retired and CPI expresses their interplay and reduces to the total number of clockticks required. Thus, this product is a quantity that directly relates to the separately measured sim time. Indeed, the comparison of this measure, depicted in Figure 5, with Figure 4 shows that the product qualitatively explains the change in sim time. While CPI increases beyond the baseline, the growth in sim time is slowed down by having fewer instructions in total.

## 6. Discussion

Our investigation characterizes the dominance of the spike-delivery phase in a weak-scaling scenario for a typical random network model (Section 2.4). At small to medium network sizes, spike delivery is the sole major contributor to simulation time. Only if thousands of compute nodes are involved, communication between nodes becomes prominent (Figure 3) while spike delivery remains the largest contributor (Jordan et al., 2018). The absolute time spent on spike delivery is dominating for small networks and grows with increasing network size. The reason for this is that the random network under study takes into account that in the mammalian brain a neuron can send spikes to more than ten-thousand targets. With increasing network size, these targets are distributed over more and more compute nodes until in the limit a neuron either finds a single target on a given compute node or more likely none at all. As the number of synapses a compute node represents is invariant under weak scaling, the node needs to process an increasing number of spikes from different source neurons. For the simulation parameters in this study, the expected number of unique source neurons and thereby absolute costs approach the limit when the network is distributed across thousands of compute nodes (Section 2.1) thus also limiting the costs of spike delivery.

In spike delivery, a thread inspects all spikes arriving at the compute node. If the thread hosts at least one target neuron of a spike, the thread needs to access a three-dimensional data structure (Figure 2) to activate the corresponding synapses and ultimately under consideration of synapse specific delays place the spike in the ring buffers of the target neurons. The present work investigates whether an alternative algorithm can reduce the number of instructions and decisions when handling an individual spike. The hypothesis is that a more compact code and more predictable control flow allows modern processors a faster execution. On purpose, no attempt is made to apply techniques like hardware prefetching or software pipelining to conceptually separate improvements of the logic of the algorithm from further optimizations that may have a stronger processor dependence. Nevertheless, we hope that the insights of the present work provide the basis for any future exploration of these issues.

The first step in our reconsideration of the spike-delivery algorithm is to look at the initial identification of the relevant spikes for each thread. Originally, each thread inspects all spikes. This means that the algorithms perform many read operations on spikes without further actions and that their proportion increases with an increasing number of threads per compute node. The alternative algorithm which we refer to as SRR (Section 4.1) carries out a partial sorting of the spikes. Each thread is responsible for an equally sized chunk of the incoming spikes and sorts them into a data structure according to the thread on which the target neuron resides and according to the type of the target synapse. Once all threads have completed their work, they find a data structure containing only relevant spikes and complete the spike delivery entirely independently from the other threads. This already leads to a reduction of simulation time between 10 and 20 % on the three computer systems tested while the detailed development of this fraction differs with network size.

As a second step, we remove an indirection originating in the initial object oriented design of the simulation code. Following the concept of describing entities of nature by software objects, neurons became objects receiving and emitting spikes and neuronal spike ring buffers an implementation detail of no relevance for other components. As a consequence when a neuron object receives a spike it needs to decide in which ring buffer to place the spike, for example, to separate excitatory from inhibitory inputs, and delegate this task to the respective buffer. Our alternative algorithm (P2RB, Section 4.2) exposes the corresponding spike ring buffer to the synapse at the time of network construction. The synapse stores the direct pointer and no further decision is required during simulation. This change further reduces simulation time by 10 to 20 %.

One computer system (JURECA CM) shows a pronounced decline in the computational advantage of the combined new algorithm (SRR+P2RB) for large network sizes, which in the case of P2RB, we assume to be due to an increase in synaptic memory footprint. An additional optimization removing a generic function call that enriches spike events by information on the identity of the source neuron mitigates the loss in performance. As this functionality is not required for the interaction between neurons, we moved the function to a more specialized part of the code (noSSG, Section 5.1).

The achievement of the combined algorithm (SRR+P2RB+noSSG) needs to be judged in light of the potential maximum gain. For small networks, spike delivery consumes 70 to 80 % of simulation time, depending on the computer system, while this relative contribution declines with growing network sizes as communication becomes more prominent. Thus, the streamlined processing of spikes reduces spike delivery by 50 % largely independent of network size. In conclusion, with the new algorithm, spike delivery still substantially contributes to simulation time.

In the small to medium scale regime (DEEP-EST CM, JURECA CM), the new code gains its superiority from executing only half of the number of instructions of the original implementation (Section 5.2). The reduction becomes slightly larger with increasing network size. This is plausible as for a given thread, the algorithm avoids processing a growing number of irrelevant spikes (SRR). As the number of synapses per compute node is fixed, but neurons have a decreasing number of targets per compute node, the number of relevant spikes increases. Therefore, decreasing the number of function calls per spike has an increasing benefit.

The picture is less clear for the average number of clockticks required to complete an instruction (Section 5.2). For small networks, the new algorithms exhibit an advantage. However, with increasing network size, eventually more clockticks per instruction are required than by the original algorithm. Nevertheless, these latencies are hard to compare as the new algorithm executes only half of the instructions and may therefore put memory interfaces under larger stress. This result already indicates that methods of latency hiding may now be successful in further reducing spike-delivery time. The product of the number of instructions and the clockticks per instruction gives an estimate of the total number of clockticks required. The observed stable improvement across all network sizes confirms the direct measurements of simulation time.

Faster simulation can trivially be achieved by reducing the generality of the code or by reducing the accuracy of the simulation. While the SRR optimization does not touch the code of individual neuron or synapse models, a critical point in the P2RB optimization with regard to code generality is the replacement of the target identifier in the synapse object (Section 2.3) by a pointer to the corresponding spike ring buffer. Synaptic plasticity is the biological phenomenon by which the strength of a synapse changes in dependence on the spiking activity of the presynaptic and the postsynaptic neuron. This is one of the key mechanisms by which brains implement system-level learning. For a wide class of models of synaptic plasticity, it is sufficient to update the synaptic weight when a presynaptic spike arrives at the synapse (Morrison et al., 2008; Stapmanns et al., 2020). However, at this point in time, the synapse typically needs to inspect a state variable of the postsynaptic neuron or even retrieve the spiking history of the postsynaptic neuron since the last presynaptic spike. This information is only available in the neuron, not in the spike ring buffer. Still, generality is preserved as in the reference simulation engine (Gewaltig and Diesmann, 2007) synapses are not restricted to a single strategy for accessing the target neuron or its spike ring buffer. A static synapse can implement the P2RB idea while a plastic synapse stays with the target identifier from which the state of the neuron, as well as the spike ring buffer, can be reached. But in this way, a plastic synapse does not profit from the advantages of P2RB at all. There are two alternatives. First, the spike ring buffer can be equipped with a pointer to the target neuron. This requires an indirection in the update of the synapse but still avoids the need to select the correct ring buffer during spike delivery. Second, the synapse can store both a target identifier and a pointer to the ring buffer. This removes the indirection for the price of additional per-synapse memory usage. There are no fundamental limitations preventing us from making both solutions available to the neuroscientist *via* different synapse types. In fact, this strategy is currently in use, for example, to provide synapse types with different target identifiers either consuming less memory or requiring fewer indirections (Section 2.2), where template-based solutions prevent the duplication of entire model codes. Users can thus select the optimal synapse-type version depending on the amount of memory available. However, making multiple versions of the same model available reduces the user-friendliness of the application. A domain specific language like NESTML (Plotnikov et al., 2016) may come to the rescue here generating more compact or faster code depending on hints of the neuroscientists to the compiler. This idea could be extended to other parts of the simulation cycle where further information is required to decide on a suitable optimization.

The incoming spike events of a compute node specify the hosting thread as well as the location of the synaptic targets, but they are unsorted with respect to the hosting thread and synapse type. Nevertheless, the present work shows that the processing of spikes can be completely parallelized requiring only a single synchronization between the threads at the point where the spikes are sorted according to target thread and synapse type, which is when all spikes have been transferred from the MPI receive buffer into the novel spike-receive register. This suggests that spike delivery fully profits from a further increase in the number of threads per compute node. Although here we concentrate on compute nodes with an order of ten cores per processor, we expect that the benefits of parallelization extend to at least an order of magnitude more cores, which matches recent hardware developments. The scaling might still be limited by the structure of the spike-receive register having separate domains for each thread writing spike data from the MPI spike receive buffer to the register. If the same number of spikes is handled by more threads, the spikes are distributed to more domains of the register such that during the actual delivery from the register to the thread-local targets each thread needs to collect its spikes from more memory domains.

The local processing of a compute node is now better understood and for large networks, the communication between nodes begins to dominate simulation time already for the machines investigated here. Current chip technology is essentially two-dimensional in contrast to the three-dimensional organization of the brain and parallelization in the brain is more fine grained. Inside, a compute node technology compensates for these advantages by communication over buses. After substantially reducing the number of instructions, we see indications that memory latency is a problem when spikes from many sources need to be processed. Therefore, it remains to be seen whether techniques of latency hiding can further push the limits imposed by the von Neumann bottleneck. Any neuromorphic hardware based on compute nodes communicating by a collective spike exchange in fixed time intervals needs to organize routing of the spikes to target neurons. The ideas presented in the present study on streamlining this process by partial parallel sorting may help in the design of adequate hardware support. However, between compute nodes, the latency of state-of-the-art inter-node communication fabrics is likely to be the next limiting factor for simulation. A possible approach to mitigate this problem is the design of dedicated neuromorphic hardware explicitly optimized for communication. The SpiNNaker project (Furber et al., 2013; Furber and Bogdan, 2020), for example, follows an extreme approach by routing packets with individual spikes to the respective processing units.

Part of the improvements in performance this study achieved come at the price of an increase in the number of lines of code and an increase in code complexity. In general, one needs to weigh the achieved performance improvements against detrimental effects on maintainability. This is particularly relevant for a community code like the one under consideration, in which experienced developers are continuously replaced by new contributors. Highly optimized code may be more difficult to keep up to date and adjust to future compute node architectures. Next to conceptual documentation of optimization to core algorithms, code generation, as explored in the NESTML project (Plotnikov et al., 2016), may be come part of a strategy to reduce this friction between performance and code accessibility. A domain specific language lets a spectrum of users concentrate on the formal description of the problem while experienced developers make sure the generator produces optimized code, possibly even adapted to specific target architectures. Until simulations are fast enough to enable the investigation of plastic networks at natural density we have to find ways to cope with increasing complexity of the algorithms and their respective implementations.

Over the last two decades, studies on simulation technology for spiking neuronal networks regularly report improvements in simulation speed on the order of several percent and improved scaling compared to the state-of-the-art technology. The stream of publications on simulation technology in the field shows that there was and still is room for substantial improvements. Nevertheless, at first sight, it seems implausible that over this time span no canonical algorithm has emerged and progress shows no sign of saturation. The solution to this riddle is that new articles tend to immediately concentrate on the latest available hardware and are interested in their limits in terms of network size. This is driven by the desire of neuroscience to overcome the limitations of extremely downscaled models and arrive at a technology capable of representing relevant parts of the brain. Moreover, investigations of novel models in computational neuroscience have a life-cycle of roughly 5 years, the same time scale at which supercomputers are installed and decommissioned. Thus, both representative network models and the hardware to simulate them are in flux, which makes comprehensive performance studies difficult. The software evolution of spiking network simulation code is largely unknown and the community may profit from a review exposing dead ends and volatile locations of the algorithm. For more systematic monitoring of technological progress, the community needs to learn how to establish and maintain reference models and keep track of benchmarking data and their respective metadata.

The present study streamlines the routing of spikes in a compute node by a fully parallel partial sorting of incoming spikes and refactoring of the code. This halves the number of instructions for this phase of the simulation and leads to a substantial reduction in simulation time. We expect that our work provides the basis for the successful application of techniques of latency hiding and vectorization.

## Data Availability Statement

All datasets and code of this study are available in a public repository (Pronold et al., 2021).

## Author Contributions

All authors listed have made a substantial, direct, and intellectual contribution to the work and approved it for publication.

## Funding

Partly supported by the European Union's Horizon 2020 (H2020) funding framework under grant agreement no. 785907 (Human Brain Project, HBP SGA2), no. 945539 (HBP SGA3), and no. 754304 (DEEP-EST), the Helmholtz Association Initiative and Networking Fund under project number SO-092 (Advanced Computing Architectures, ACA), and the Deutsche Forschungsgemeinschaft (DFG, German Research Foundation) -368482240/GRK2416. The use of the JURECA supercomputer in Jülich was made possible through VSR computation time grant Brain-Scale Simulations JINB33. This research used resources of K computer at the RIKEN Advanced Institute for Computational Science. Supported by the project Exploratory Challenge on Post-K Computer (Understanding the neural mechanisms of thoughts and its applications to AI) of the Ministry of Education, Culture, Sports, Science and Technology (MEXT).

## Conflict of Interest

The authors declare that the research was conducted in the absence of any commercial or financial relationships that could be construed as a potential conflict of interest.

## Publisher's Note

All claims expressed in this article are solely those of the authors and do not necessarily represent those of their affiliated organizations, or those of the publisher, the editors and the reviewers. Any product that may be evaluated in this article, or claim that may be made by its manufacturer, is not guaranteed or endorsed by the publisher.

## Footnotes

^1^ https://www.nest-simulator.org

^2^ https://nest-simulator.readthedocs.io

^3^ https://www.deep-projects.eu

^4^ https://software.intel.com/vtune

^5^ https://www.fz-juelich.de/jsc/jube
